# Alteration of the steroidogenesis in boys with autism spectrum disorders

**DOI:** 10.1038/s41398-020-01017-8

**Published:** 2020-10-06

**Authors:** Katarína Janšáková, Martin Hill, Diana Čelárová, Hana Celušáková, Gabriela Repiská, Marie Bičíková, Ludmila Máčová, Daniela Ostatníková

**Affiliations:** 1 Institute of Physiology, Faculty of Medicine, Comenius University in Bratislava, Bratislava, Slovak Republic; 2 Department of Steroid Hormones and Proteohormones, Institute of Endocrinology, Prague, Czech Republic

**Keywords:** Molecular neuroscience, Diagnostic markers

## Abstract

The etiology of autism spectrum disorders (ASD) remains unknown, but associations between prenatal hormonal changes and ASD risk were found. The consequences of these changes on the steroidogenesis during a postnatal development are not yet well known. The aim of this study was to analyze the steroid metabolic pathway in prepubertal ASD and neurotypical boys. Plasma samples were collected from 62 prepubertal ASD boys and 24 age and sex-matched controls (CTRL). Eighty-two biomarkers of steroidogenesis were detected using gas-chromatography tandem-mass spectrometry. We observed changes across the whole alternative backdoor pathway of androgens synthesis toward lower level in ASD group. Our data indicate suppressed production of pregnenolone sulfate at augmented activities of CYP17A1 and SULT2A1 and reduced HSD3B2 activity in ASD group which is partly consistent with the results reported in older children, in whom the adrenal *zona reticularis* significantly influences the steroid levels. Furthermore, we detected the suppressed activity of CYP7B1 enzyme readily metabolizing the precursors of sex hormones on one hand but increased anti-glucocorticoid effect of 7α-hydroxy-DHEA via competition with cortisone for HSD11B1 on the other. The multivariate model found significant correlations between behavioral indices and circulating steroids. From dependent variables, the best correlation was found for the social interaction (28.5%). Observed changes give a space for their utilization as biomarkers while reveal the etiopathogenesis of ASD. The aforementioned data indicate a direction of the future research with a focus on the expression and functioning of genes associated with important steroidogenic enzymes in ASD patients from early childhood to adrenarche.

## Introduction

Autism spectrum disorders (ASD) represent a group of neurodevelopmental disorders with multifactorial etiopathogenesis. Several theories have been proposed regarding the cause of autism but no one has yet been definitely proven or disproved^1–3^. One of them is the extreme male brain theory describing ASD cognitive traits in toward to a male behavioral pattern in a sense of dominance of systemizing as a male feature at the expense of empathizing as a female feature^1^. This theory together with the fetal androgen theory of autism^4^ tries to find the cause of ASD in prenatal exposure to male steroid hormones dominantly testosterone (TST). Since TST plays an important role during early developmental stages of brain^5^, thus, these theories assume it might contribute to the development of autistic traits^1^. Moreover, this theory is supported by the fact that ASD is diagnosed in male individuals four times more likely than in females^6^. Studies performed on amniotic fluid found altered hormonal profile in a sense of higher level of androgens in children who turned to have ASD^7,8^. Active androgens are synthesized via two alternative pathways. The first of them is known as the classic “frontdoor” pathway with pregnenolone serving as androgen precursor, which underwent a conversion to DHEA and subsequently to androstenediol. These metabolic steps are catalyzed by CYP17A1 (in the C17,20-lyase step) and (mostly adrenal) AKR1C3 enzyme, respectively. DHEA and androstenediol are readily sulfated by SULT2A1 in adrenal cortex and their sulfates serves as the stock pool for the production of active androgens of the adrenal origin as the production of androgens in early childhood of boys is limited to extra-gonadal tissues, such as adrenal, skin, etc. These sulfated primary androgens, may be subsequently deconjugated, and metabolized by HSD3B1 and HSD3B2 isoforms to androstenedione and TST and then to 5α/β-reduced 17-oxo- and 17β-androgens, respectively. In addition, the androstenedione may be readily converted to testosterone by adrenal AKR1C3. From the aforementioned substances, TST, 5α-dihydrotestosterone, and 11-oxo-testosterone are known as the most potent bioactive androgens. Besides the “frontdoor” pathway the dihydrotestosterone may be also formed by so called “backdoor” pathway. This pathway is based on a direct conversion of 5α/β-reduced pregnane steroids (C21) to their 5α/β-reduced androgen (C19) metabolites which is catalyzed by the same enzyme converting pregnenolone to DHEA (CYP17A1 in the C17,20-lyase step). These 5α/β-reduced androgen (C19) metabolites include also the most active androgen 5α-dihydrotestosterone. The “backdoor” pathway is crucial for androgen synthesis in marsupials but may also be active in various human steroid-related disorders^9^. The extragonadal androgen synthesis via the “frontdoor” pathway primarily proceeds in adrenal *zona reticularis* (ZR), however, like the testicular activity, the ZR functioning is minor in early childhood^10^.

Contrary, studies assessing postnatal androgen levels in ASD are indecisive. While some of them report high amount of androgens in ASD (mainly TST) other studies claim no changes, effect or relation of postnatal levels of various sex steroids on ASD^11,12^. Higher level of androgens were observed in saliva of prepubertal ASD boys and girls^13^ as well as in urine of pubertal ASD boys compared to controls^12^. Adult ASD males showed no differences in serum TST while its concentration in ASD women was higher compared to matched controls^14^. In addition, no association between autistic traits assessed using the Autism-spectrum Quotient questionnaire (AQ) and salivary TST concentration in adult males were observed^15^.

While research is primarily focused on sex hormones, the role of other steroids like glucocorticoids or mineralocorticoids in the pathogenesis of ASD does not often come under scrutiny. The impact of these hormones in the complexity and continuity of steroidal pathway^16,17^, especially in relation to ASD is not fully described. The aim of this study was to analyze steroid metabolic pathway in plasma of male children diagnosed with ASD and healthy neurotypical controls.

## Materials and methods

This study was approved by the Ethical committee of the Faculty of Medicine, Comenius University, and the University Hospital in Bratislava, Slovakia and it is consistent with the 1964 Helsinki declaration and its later amendments.

Parents were aware of design of the study and the informed consent form was signed by both (if available) parents or caregivers of corresponding child.

### Diagnostics of autism

Children suspected for the presence of autism spectrum disorder (ASD) by a pediatrician were recruited and diagnosed in Academic research center for autism, Faculty of Medicine, Comenius University in Bratislava. Children were diagnosed according to Diagnostic and Statistical Manual of Mental Disorders 5^th^ edition (DSM-5)^18^. The diagnostic process was comprised of the two diagnostic methods Autism Diagnostic Observation Schedule 2nd revision (ADOS-2)^19^ and Autism Diagnostic Interview-Revised (ADI-R)^20^ which are considered to be “gold standard” diagnostic tools for ASD assessment. Diagnostic procedures were performed by trained clinical psychologists and the decision was made after consensus in clinical judgment. Children who did not meet the described criteria despite their social and communication impairment were not included into the study.

ADOS-2 represents a standardized behavioral observation of ASD symptoms suitable for individuals of 12 months through adulthood on different developmental levels and language skills. It comprises of various structured and non-structured situations in which social, communicative and other behaviors relevant for ASD diagnosis are observed.

Diagnostics of ASD using ADI-R is an interview with the parent or a caregiver of a child. It follows the developmental history of the individual as well as presence of the most significant behaviors related to ASD diagnosis. ADI-R diagnostic tool provides categorical results for three following subdomains: quality of social interaction (A); communication and language (B); and repetitive, restricted and stereotyped interests and behavior (C).

### Participants and sample collection

A total number of 86 pre-pubertal, preschool boys were included into the study. Sixty-two individuals were children diagnosed with autism (ASD, 4.4 ± 1.1 years) in module 1 and 24 age and sex-matched neurotypical controls (CTRL, 4.29 ± 0.95 years). Control children were chosen by pediatrician based on no history of ASD or other neurodevelopmental disorder.

Exclusion criteria for recruitment were as follows: presence of a systemic disease, acute illness, using antibiotics or steroidal and non-steroidal drugs and present any other psychiatric disorder except ASD.

Blood samples were collected immediately after ADOS-2 diagnostic procedure into sterile EDTA tubes (Sarstedt, Nümbrecht, Germany) around 10 am. Blood taken from controls was collected at the same time. All samples were delivered into the laboratory immediately after collection and centrifuged at 3000 × *g*, 4 °C for 10 min. Plasma samples were stored at −80 °C until the analysis.

### ADOS-2, Module 1 description

Module 1 is selected on the basis of expressive language level and age and is intended for individuals who do not consistently use phrase speech and who are more than 31 months old. This module comprises of ten tasks, during which the administrator provides presses for social interaction and communication. Immediately after administering all tasks, the professional assigns the codes for each of the observed behaviors according to the diagnostic algorithm. After assigning codes to the behaviors, two separate domains can be calculated: social affect and restricted and repetitive behaviors in addition to overall total ADOS-2 score, which combines scores of these domains.

### Steroid analysis

All steroids and their polar conjugates were analyzed using gas-chromatography tandem-mass spectrometry (GC-MS/MS). Whole procedure was performed according to Hill et al.^21^.

### Statistical analysis

#### Age-adjusted linear model

Statgraphic centurion XV statistical software (Statpoint, Inc., Herndon, Virginia, USA) was used for this analysis. Linear model adjusted to constant age was used for the separation of variability in the dependent variable shared with age from the one explained by the health status i.e., ASD vs. CTRL. Data were transformed using a power transformation for achieving the homoscedasticity and symmetry of the data prior to their further processing. Residual analysis was used for the checking of the homogeneity and the distribution of the transformed data.

#### Multivariate regression with a reduction of a dimensionality, orthogonal projections to latent structure (OPLS/O2PLS)

SIMCA-P s12.0. (Umetrics AB, Umeå, Sweden) was used for the processing of the obtained data. OPLS/O2PLS was used for the assessment of the relationship between the analyzed steroids and ASD or behavioral parameters.

As a single dependent variable, the logarithm of the likelihood ratio that the subject is an individual having ASD to the probability that individual is a neurotypical control was chosen. Age of the ASD and CTRL individuals together with the concentrations of steroids were considered as predictors. The variability of these predictors was divided into two groups of mutually independent components. First, variability predictors related to the probability of the presence of the ASD disorder as a predictive component. Second, orthogonal components explaining the variability shared within the highly intercorrelated predictors. The relevant predictors were chosen via variable important statistics.

#### Multiple regression

Like the multivariate regression the ordinary least squares multiple regression was completed using the SIMCA software. This analysis approach was used without the reduction of dimensionality.

In all analyses, *p* value below 0.05 was considered as significant. Data are presented as regression coefficients with their *t*-statistics (the ratio of regression coefficient to its standard error).

## Results

Circulating unconjugated and conjugated steroids were detected in plasma of children diagnosed with autism categorized in module 1, ASD, and age-matched neurotypical controls, CTRL.

The relationship between two independent variables i.e., group represented by CTRL or ASD and age and 83 individual potential predictors representing the hormones of steroid metabolic pathways were detected. Age-adjusted ANCOVA model revealed a significant difference in 20 steroids.

Significantly lower level of pregnenolone sulfate was observed in ASD compared to CTRL (*F* = 4.4, *p* = 0.040). Significantly lower level of progesterone was observed in ASD compare to CTRL (*F* = 7.6, *p* = 0.007). Metabolites of progesterone, 16α-hydroxyprogesterone (*F* = 7.7, *p* = 0.007), 20α-dihydroprogesterone (*F* = 4.2, *p* = 0.024) and cortisone (*F* = 6, *p* = 0.019) were lower in ASD compared to CTRL. Similarly, conjugated epipregnanolone, conjugated 5α-pregnane-3α,20α-diol (*F* = 10.3, *p* = 0.002), 5β-pregnane-3β,20α-diol (*F* = 9.3, *p* = 0.003), conjugated 5α-pregnane-3β,17,20α-triol (*F* = 32.4, *p* < 0.001) and conjugated 5β-pregnane-3α,17,20α-triol (*F* = 7.4, *p* = 0.009) were also significantly lower in ASD compared to CTRL. All C19 steroids showing significant differences between ASD and CTRL were lower in ASD. This concern 7α-hydroxy-DHEA (*F* = 4.5, *p* = 0.037), 7-oxo-DHEA (*F* = 17.2, *p* < 0.001), conjugated 5-androstene-3β,16α,17β-triol (*F* = 16.1, *p* < 0.001), androsterone sulfate (*F* = 7.5, *p* = 0.008), epiandrosterone sulfate (*F* = 6.7, *p* = 0.012), etiocholanolone (3α,5β-THA) sulfate (*F* = 6.8, *p* = 0.011), epietiocholanolone sulfate (*F* = 12.7, *p* < 0.001), conjugated 5α-androstane-3β,17β-diol (*F* = 9.6, *p* = 0.004), 5β-androstane-3β,17β-diol (*F* = 9.6, *p* = 0.004), and 11β-hydroxyetiocholanolone (*F* = 9.7, *p* = 0.003). All concentrations are presented as a mean together with lower and upper 95% confidence interval in Table 1 for every marker individually. There were many markers showing trends and marginally non-significant differences between CTRL and ASD. Thus, results from these markers with *p* value for the factor Group up to *p* = 0.2 are presented in the Table 1 as well while the results with *p* value above *p* = 0.2 are not shown. It is obvious that these markers create a line from pregnenolone via a first important point of steroidogenesis, progesterone and its metabolites 20α-dihydroprogesterone and its conjugate and 16α-hydroxyprogesterone. This line is heading toward cortisol. Next changes might be seen in the metabolic pathway of progesterone via conjugated epipregnanolone, conjugated 5α-pregnane-3α,20α-diol, 5β-pregnane-3β,20α-diol, conjugated 5α-pregnane-3β,17,20α-triol, conjugated 5β-pregnane-3α,17,20α-triol. Reduced pregnanes may serve as precursors for formation of reduced androstanes, including androsterone or the most active androgen 5α-dihydrotestosterone via “backdoor” pathway. The changes in metabolic pathway of androgens were seen around the metabolism of DHEA, specifically its metabolites 7α-hydroxy/7-oxo-DHEA and conjugated 5-androsten-3β,16α,17β-triol. Androstanes crossing the metabolism of androgens like 11β-hydroxyetiocholanolone, conjugated 5α-androstane-3β,17β-diol and conjugated 5β-androstane-3β,17β-diol as well as sulfates of reduced 17-oxo androstanes, such as androsterone, epiandrosterone, etiocholanolone, and epietiocholanolone showed changes as well.

**Table 1:** Table showing concentrations of unconjugated and conjugated/sulfated circulating steroids in CTRL and ASD individuals with statistical significance up to *p* = 0.2.

			Mean [µmol/l] (lower; upper 95% CI)		ANCOVA (factor ASD and covariate Age) *F* = *F*-value, *p* = p-value
	Steroid	Abbreviation	CTRL	ASD	
C21∆^5^	Pregnenolone	Preg	0.799 (0.652, 0.977)	0.603 (0.525, 0.692)	ASD: *F* = 2.6, *p* = 0.111; Age: *F* = 0.1, *p* = 0.748
	Pregnenolone sulfate	PregC	55 (46.2, 65.7)	40 (35.4, 45.2)	ASD: *F* = 4.4, ***p*** = **0.04**; Age: *F* = 11.7, ***p*** = **0.001**
	20α-Dihydropregnenolone	Preg20a	0.585 (0.491, 0.7)	0.475 (0.422, 0.536)	ASD: *F* = 1.9, *p* = 0.171; Age: *F* = 1.5, *p* = 0.221
	20α-Dihydropregnenolone sulfate	Preg20aC	168 (141, 202)	136 (121, 153)	ASD: *F* = 2, *p* = 0.164; Age: *F* = 0.2, *p* = 0.639
C19∆^5^	DHEA	DHEA	1.49 (1.29, 1.73)	1.21 (1.11, 1.33)	ASD: *F* = 3, *p* = 0.088; Age: *F* = 2.5, *p* = 0.117
	DHEA sulfate	DHEAC	159 (117, 217)	105 (85.7, 130)	ASD: *F* = 2.5, *p* = 0.12; Age: *F* = 10.6, ***p*** = **0.002**
	7α-Hydroxy-DHEA	DHEA7a	0.322 (0.241, 0.423)	0.188 (0.151, 0.231)	ASD: *F* = 4.5, ***p*** = **0.037**; Age: *F* = 4.6, ***p*** = **0.035**
	7-oxo-DHEA	DHEA7o	0.207 (0.171, 0.25)	0.103 (0.0906, 0.118)	ASD: *F* = 17.2, ***p*** < **0.001**; Age: *F* = 4.5, ***p*** = **0.038**
	Androstenediol	Adiol	0.298 (0.247, 0.36)	0.219 (0.194, 0.248)	ASD: *F* = 3.8, *p* = 0.056; Age: *F* = 1.6, *p* = 0.212
	Androstenediol sulfate	AdiolC	26.2 (18.4, 37.7)	15.4 (12.3, 19.3)	ASD: *F* = 3.2, *p* = 0.079; Age: *F* = 1.6, *p* = 0.213
	Conjugated 5-androstene-3β,16α,17β-triol	AT16aC	11.4 (9.37, 13.8)	5.69 (4.93, 6.54)	ASD: *F* = 16.1, ***p*** < **0.001**; Age: *F* = 15.5, ***p*** < **0.001**
C21∆^4^	Progesterone	Prog	0.131 (0.103, 0.166)	0.0725 (0.0607, 0.0864)	ASD: *F* = 7.6, ***p*** = **0.007**; Age: *F* = 2.4, *p* = 0.125
	17-Hydroxyprogesterone	Prog17	0.606 (0.508, 0.723)	0.484 (0.431, 0.544)	ASD: *F* = 2.3, *p* = 0.138; Age: *F* = 15.5, ***p*** < **0.001**
	16α-Hydroxyprogesterone	Prog16a	0.543 (0.436, 0.678)	0.323 (0.28, 0.374)	ASD: *F* = 7.7, ***p*** = **0.007**; Age: *F* = 0.3, *p* = 0.561
	20α-Dihydroprogesterone	Prog20a	0.105 (0.0906, 0.123)	0.0788 (0.0718, 0.0868)	ASD: *F* = 5.3, ***p*** = **0.024**; Age: *F* = 0.3, *p* = 0.575
	Cortisone	E	120 (105, 138)	90.9 (83.6, 99.2)	ASD: *F* = 6, ***p*** = **0.019**; Age: *F* = 0.2, *p* = 0.655
C21 5α/β- reduced	Conjugated 20α-dihydroprogesterone	Prog20aC	0.843 (0.733, 0.971)	0.677 (0.617, 0.743)	ASD: *F* = 3.4, *p* = 0.07; Age: *F* = 6.5, ***p*** = **0.013**
	Isopregnanolone (3β5α-THP)	P3b5a	0.126 (0.0969, 0.164)	0.0931 (0.0779, 0.111)	ASD: *F* = 1.8, *p* = 0.182; Age: *F* = 4.4, ***p*** = **0.039**
	Isopregnanolone (3β5α-THP) sulfate	P3b5aC	5.15 (4.57, 5.79)	4.3 (3.94, 4.68)	ASD: *F* = 3, *p* = 0.087; Age: *F* = 0.7, *p* = 0.402
	Conjugated epipregnanolone (3β5β-THP)	P3b5bC	1.12 (0.974, 1.28)	0.873 (0.785, 0.967)	ASD: *F* = 4.1, ***p*** = **0.048**; Age: *F* = 1.7, *p* = 0.197
	Conjugated 5α,20α-tetrahydroprogesterone	P5a20aC	0.333 (0.243, 0.462)	0.225 (0.185, 0.276)	ASD: *F* = 2.2, *p* = 0.144; Age: *F* = 1.2, *p* = 0.277
	Conjugated 5α-pregnane-3α,20α-diol	P3a5a20aC	9.39 (8.05, 10.9)	6.19 (5.58, 6.85)	ASD: *F* = 10.3, ***p*** = **0.002**; Age: *F* = 2.5, *p* = 0.119
	Conjugated 5α-pregnane-3β,20α-diol	P3b5a20aC	156 (131, 186)	120 (108, 134)	ASD: *F* = 3.3, *p* = 0.071; Age: *F* = 0.6, *p* = 0.45
	Conjugated 5β-pregnane-3α,20α-diol	P3a5b20aC	2.13 (1.83, 2.47)	1.79 (1.61, 1.99)	ASD: *F* = 1.8, *p* = 0.182; Age: *F* = 1.8, *p* = 0.182
	5β-Pregnane-3β,20α-diol	P3b5b20a	0.269 (0.199, 0.366)	0.124 (0.103, 0.15)	ASD: *F* = 9.3, ***p*** = **0.003**; Age: *F* = 4.6, ***p*** = **0.036**
	17-Hydroxyallopregnanolone sulfate	P3a5a17C	1.77 (1.47, 2.11)	2.17 (1.95, 2.41)	ASD: *F* = 2, *p* = 0.167; Age: *F* = 0, *p* = 0.959
	17-Hydroxypregnanolone	P3a5b17	0.0169 (0.0141, 0.0204)	0.0209 (0.0185, 0.0237)	ASD: *F* = 1.8, *p* = 0.185; Age: *F* = 81.6, ***p*** < **0.001**
	5α-Pregnane-3α,17,20α-triol	P3a5a17a20a	0.55 (0.414, 0.711)	0.375 (0.293, 0.471)	ASD: *F* = 2.2, *p* = 0.147; Age: *F* = 1.5, *p* = 0.223
	Conjugated 5α-pregnane-3β,17,20α-triol	P3b5a17a20aC	4.81 (3.82, 6.02)	1.52 (1.28, 1.79)	ASD: *F* = 32.4, ***p*** < **0.001**; Age: *F* = 10.6, ***p*** = **0.002**
	5β-Pregnane-3α,17,20α-triol	P3a5b17a20a	0.221 (0.182, 0.266)	0.275 (0.243, 0.31)	ASD: *F* = 1.9, *p* = 0.171; Age: *F* = 7.4, ***p*** = **0.009**
	Conjugated 5β-pregnane-3α,17,20α-triol	P3a5b17a20aC	26.3 (21.7, 31.9)	17 (15, 19.2)	ASD: *F* = 7.4, ***p*** = **0.009**; Age: *F* = 25.2, ***p*** < **0.001**
C19 5α/β- reduced	Androsterone (3α5α-THA) sulfate	A3a5aC	75.4 (58.4, 96.8)	41.8 (35.5, 49.1)	ASD: *F* = 7.5, ***p*** = **0.008**; Age: *F* = 27.9, ***p*** < **0.001**
	Epiandrosterone (3β5α-THA) sulfate	A3b5aC	23.1 (18.1, 29.6)	13.3 (11.2, 15.8)	ASD: *F* = 6.7, ***p*** = **0.012**; Age: *F* = 8.5, ***p*** = **0.005**
	Etiocholanolone (3α5β-THA) sulfate	A3a5bC	2.88 (2.51, 3.3)	2.13 (1.96, 2.32)	ASD: *F* = 6.8, ***p*** = **0.011**; Age: *F* = 37.8, ***p*** < **0.001**
	Epietiocholanolone (3β5β-THA)	A3b5b	0.0325 (0.0249, 0.0419)	0.0229 (0.0191, 0.0272)	ASD: *F* = 2.4, *p* = 0.125; Age: *F* = 2.4, *p* = 0.13
	Epietiocholanolone (3β5β-THA) sulfate	A3b5bC	2.47 (2.02, 3.03)	1.37 (1.22, 1.55)	ASD: *F* = 12.7, ***p*** < **0.001**; Age: *F* = 25.2, ***p*** < **0.001**
	5α-Androstane-3α,17β-diol	A3a5a17b	0.0761 (0.0677, 0.0862)	0.0646 (0.0597, 0.07)	ASD: *F* = 2.7, *p* = 0.109; Age: *F* = 0.8, *p* = 0.389
	Conjugated 5α-androstane-3β,17β-diol	A3b5a17bC	2.43 (1.96, 3.01)	1.62 (1.43, 1.84)	ASD: *F* = 5.3, ***p*** = **0.026**; Age: *F* = 9, ***p*** = **0.004**
	Conjugated 5β-androstane-3α,17β-diol	A3a5b17bC	0.248 (0.172, 0.366)	0.158 (0.129, 0.195)	ASD: *F* = 2.3, *p* = 0.133; Age: *F* = 0.6, *p* = 0.434
	5β-Androstane-3β,17β-diol	A3b5b17b	0.0667 (0.0519, 0.0844)	0.0316 (0.0247, 0.0398)	ASD: *F* = 9.6, ***p*** = **0.004**; Age: *F* = 0.1, *p* = 0.784
	Conjugated 5β-androstane-3β,17β-diol	A3b5b17bC	0.0944 (0.0641, 0.14)	0.0525 (0.0421, 0.0658)	ASD: *F* = 3.4, *p* = 0.071; Age: *F* = 2.1, *p* = 0.151
	11β-Hydroxyandrostenedione	A211b	87.9 (73.7, 103)	109 (99, 119)	ASD: *F* = 2.6, *p* = 0.111; Age: *F* = 4.9, ***p*** = **0.03**
	11β-Hydroxyetiocholanolone	A3a5b11b	6.26 (5.58, 7.01)	4.61 (4.26, 4.98)	ASD: *F* = 9.7, ***p*** = **0.003**; Age: *F* = 0.1, *p* = 0.738
	11β-Etiocholanolone sulfate	A3a5b11bC	3.58 (2.73, 4.61)	2.27 (1.89, 2.72)	ASD: *F* = 3.8, *p* = 0.055; Age: *F* = 4.5, ***p*** = **0.036**

All data are presented as mean together with lower and upper 95% confidence interval; age-adjusted linear model was used for evaluation of differences between CTRL and ASD. Bold values denote significant differences between the analysed groups.

*AS*D autism spectrum disorder, *CI* confidence interval, *CTRL* control.

**Fig. 1:**
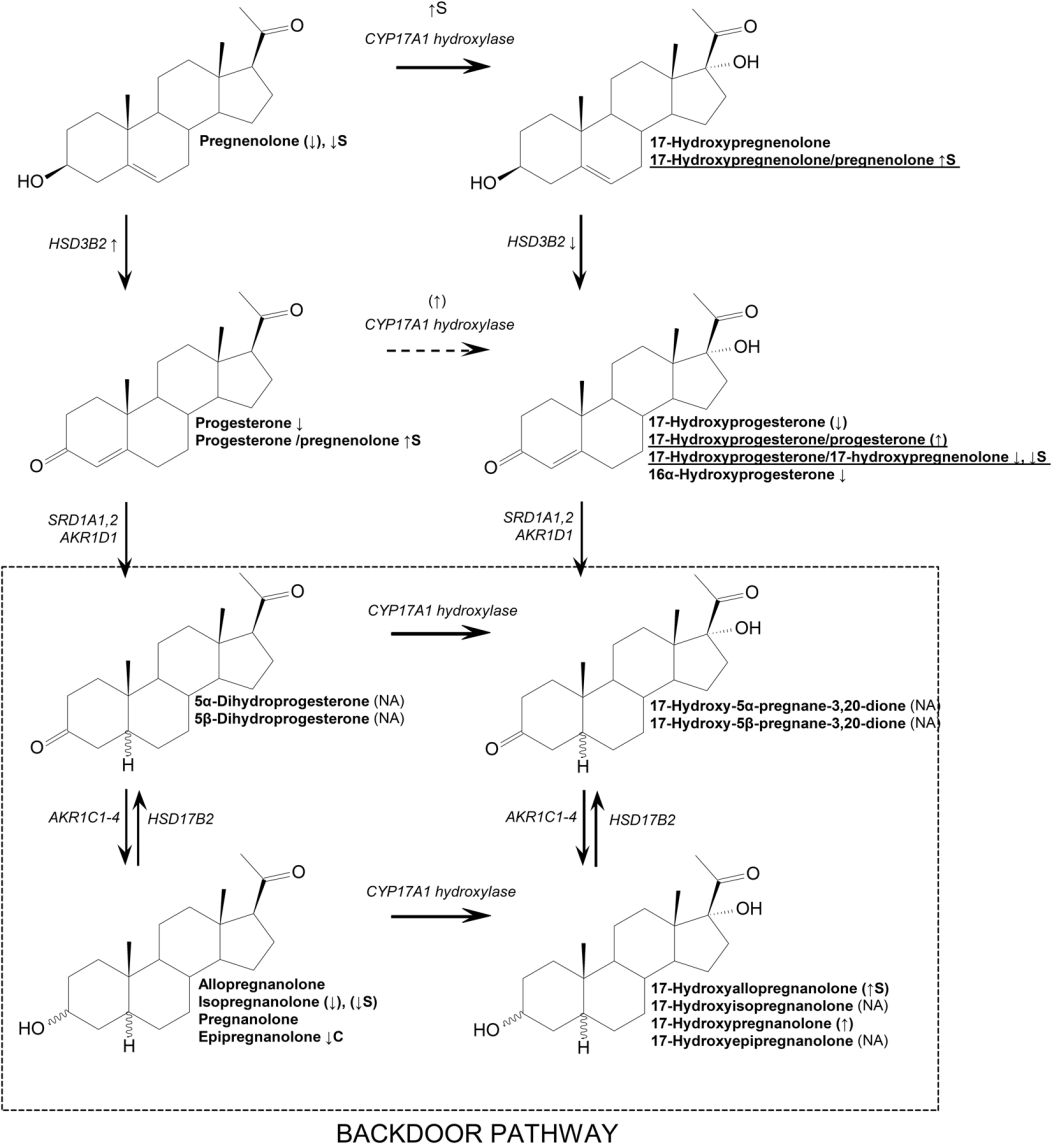
Simplified scheme illustrating differences between CTRL and ASD groups for CYP17A1 17-hydroxylase step. ↑/↓ denotes higher/lower steroid or steroid ratio in ASD group compare to control group; ASD, autism spectrum disorder; C, conjugate; CTRL, control; S, sulfate; NA, hormone not assessed; (↑/↓), non-significant difference ↑/↓ significant difference between CTRL and ASD in favor to ASD with *p* value up to *p* = 0.2 according to the results presented in Tables 1 and  2.

The ratio of 17-hydroxypregnenolone/pregnenolone sulfates which conversion is secured by CYP17A1 hydroxylase showed significant differences toward the higher level in ASD compared to CTRL (*F* = 4.6, *p* = 0.035). Same, higher cortisol/corticosterone ratio was higher in ASD compared to CTRL (*F* = 4.8, *p* = 0.035) (Table 2, Fig. 1).

**Table 2:** Age-adjusted differences between controls and ASD patients for product to precursor ratios reflecting CYP17A1 activity in the 17-hydroxylase metabolic step, CYP17A1 activity in the C17,20-lyase metabolic step, ratios of conjugated to unconjugated steroid (C/U) reflecting a balance between sulfotransferase and sulfatase activities, steroid ratios reflecting the formation of immunomodulatory and immunoprotective steroids, product to precursor ratios reflecting HSD3B2 activity, steroid ratios of reflecting AKR1C1 activity.

		Mean (lower; upper 95% CI)		
		CTRL	ASD	ANCOVA
*Steroid ratio—17-hydroxy/17-deoxysteroid*				
17-Hydroxypregnenolone/pregnenolone, sulfates		0.0859 (0.073, 0.1)	0.117 (0.11, 0.13)	ASD: *F* = 4.6, ***p*** = **0.035**; Age: *F* = 1.1, *p* = 0.309
17-Hydroxyprogesterone/progesterone		4.97 (3.96, 6.27)	6.99 (5.96, 8.23)	ASD: *F* = 2.9, *p* = 0.094; Age: *F* = 0.7, *p* = 0.421
Cortisol/corticosterone		37.3 (30.7, 46.3)	57.3 (48.4, 68.6)	ASD: *F* = 4.8, ***p*** = **0.035**; Age: *F* = 0.6, *p* = 0.455
*Steroid ratio, C19-/C21-17-deoxy-steroid*				
DHEA/pregnenolone		1.95 (1.73, 2.2)	2.49 (2.28, 2.73)	ASD: *F* = 5.2, ***p*** = **0.026**; Age: *F* = 0.1, *p* = 0.784
DHEA sulfate/pregnenolone sulfate		2.42 (2.15, 2.75)	3.28 (3, 3.6)	ASD: *F* = 7.4, ***p*** = **0.009**; Age: *F* = 22.9, ***p*** < **0.001**
Androsterone/allopregnanolone		2.79 (2.43, 3.17)	3.92 (3.63, 4.22)	ASD: *F* = 10.9, ***p*** = **0.002**; Age: *F* = 0.4, *p* = 0.515
Androsterone/allopregnanolone, sulfates		36.3 (30.7, 42.8)	27.2 (24.5, 30.1)	ASD: *F* = 4.3, ***p*** = **0.041**; Age: *F* = 56.1, ***p*** < **0.001**
Epiandrosterone/isopregnanolone		1.82 (1.65, 2)	2.27 (2.11, 2.43)	ASD: *F* = 6.8, ***p*** = **0.012**; Age: *F* = 15, ***p*** < **0.001**
*Steroid conjugate/unconjugated steroid ratio*				
Pregnenolone, C/U		42.7 (35.8, 50.9)	70.8 (63.9, 78.4)	ASD: *F* = 12.3, ***p*** < **0.001**; Age: *F* = 0.1, *p* = 0.828
17-Hydroxypregnenolone, C/U		2.05 (1.85, 2.28)	3.13 (2.88, 3.4)	ASD: *F* = 18.3, ***p*** < **0.001**; Age: *F* = 1.7, *p* = 0.197
17,20α-Dihydroxy-4-pregnene-3-one, C/U		2.67 (2.14, 3.34)	6.22 (5.54, 6.98)	ASD: *F* = 22.9, ***p*** < **0.001**; Age: *F* = 0.8, *p* = 0.369
Allopregnanolone (3α5α-THP), C/U		30.4 (24.8, 37.2)	47.8 (41.7, 54.8)	ASD: *F* = 6.7, ***p*** = **0.012**; Age: *F* = 4, *p* = 0.051
Isopregnanolone (3β5α-THP), C/U		26.5 (21.8, 31.8)	41.6 (37, 46.5)	ASD: *F* = 8.6, ***p*** = **0.005**; Age: *F* = 1.9, *p* = 0.179
5α-Pregnane-3α,17,20α-triol, C/U		76.7 (68.8, 85.3)	102 (93.8, 110)	ASD: *F* = 8.9, ***p*** = **0.006**; Age: *F* = 12.4, ***p*** = **0.002**
5α-Pregnane-3β,17,20α-triol, C/U		34.2 (29.1, 45)	20.3 (18.3, 25.8)	ASD: *F* = 10.9, ***p*** = **0.002**; Age: *F* = 0.5, *p* = 0.467
DHEA, C/U		120 (99.2, 146)	89.9 (79.1, 102)	ASD: *F* = 3.1, *p* = 0.081; Age: *F* = 12.7, ***p*** < **0.001**
Androstenediol, C/U		77.7 (58.1, 103)	107 (88.3, 129)	ASD: *F* = 1.7, *p* = 0.192; Age: *F* = 3.5, *p* = 0.065
5α-Pregnane-3α,20α-diol, C/U		34.7 (28.6, 41.7)	22.5 (19.9, 25.5)	ASD: *F* = 6.9, ***p*** = **0.011**; Age: *F* = 7.6, ***p*** = **0.008**
5β-Pregnane-3β,20α-diol, C/U		22 (16.6, 29.3)	47.4 (39.4, 57.1)	ASD: *F* = 10, ***p*** = **0.002**; Age: *F* = 4.2, ***p*** = **0.045**
Androsterone (3α5α-THA), C/U		669 (535, 828)	265 (226, 309)	ASD: *F* = 22.1, ***p*** < **0.001**; Age: *F* = 17.5, ***p*** < **0.001**
5α-Androstane-3α,17β-diol, C/U		21.9 (15.9, 30)	14.6 (12.1, 17.6)	ASD: *F* = 2.4, *p* = 0.126; Age: *F* = 0.1, *p* = 0.771
5β-Androstane-3α,17β-diol, C/U		9.22 (5.71, 14.8)	2.44 (1.78, 3.34)	ASD: *F* = 10.8, ***p*** = **0.002**; Age: *F* = 0.8, *p* = 0.368
5β-Androstane-3β,17β-diol, C/U/		2.02 (1.36, 3.21)	4.51 (3.12, 6.47)	ASD: *F* = 4.1, *p* = 0.059; Age: *F* = 1.4, *p* = 0.262
*Enzyme*	*Steroid ratio*			
CYP7B1	7α-Hydroxy-DHEA/DHEA	0.22 (0.18, 0.26)	0.12 (0.1, 0.14)	ASD: *F* = 13, ***p*** < **0.001**; Age: *F* = 3.9, *p* = 0.052
CYP3A4	5-Androstene-3β,16α,17β-triol/androstenediol, sulfates	0.246 (0.21, 0.28)	0.426 (0.377, 0.48)	ASD: *F* = 14.1, ***p*** < **0.001**; Age: *F* = 0, *p* = 0.911
	7β-Hydroxy-DHEA/DHEA	0.0776 (0.07, 0.09)	0.0587 (0.05, 0.06)	ASD: *F* = 6.8, ***p*** = **0.012**; Age: *F* = 0.1, *p* = 0.741
HSD11B1	7-oxo-DHEA/7α-Hydroxy-DHEA	0.488 (0.38, 0.64)	0.726 (0.614, 0.86)	ASD: *F* = 3, *p* = 0.089; Age: *F* = 12.2, ***p*** < **0.001**
	7β-Hydroxy-DHEA/7α-hydroxy-DHEA	0.295 (0.25, 0.35)	0.558 (0.48, 0.65)	ASD: *F* = 13.8, ***p*** < **0.001**; Age: *F* = 2.4, *p* = 0.125
	5-Androstene-3β,7β,17β-triol/5-androstene-3β,7α,17β-triol	0.371 (0.33, 0.41)	0.304 (0.28, 0.33)	ASD: *F* = 4.1, ***p*** = **0.047**; Age: *F* = 13.1, ***p*** < **0.001**
	Cortisol/cortisone	2.88 (2.56, 3.29)	2.35 (2.2, 2.52)	ASD: *F* = 4.4, ***p*** = **0.045**; Age: *F* = 11.5, ***p*** = **0.002**
AKR1C3	Androstenediol/DHEA	0.21 (0.18, 0.23)	0.169 (0.16, 0.18)	ASD: *F* = 5.4, ***p*** = **0.024**; Age: *F* = 0.4, *p* = 0.514
	5-Androstene-3β,7α,17β-triol/7α-hydroxy-DHEA	0.197 (0.12, 0.30)	0.37 (0.273, 0.50)	ASD: *F* = 2.7, *p* = 0.106; Age: *F* = 2, *p* = 0.163
*HSD3B2*				
17-Hydroxyprogesterone/17-hydroxypregnenolone		0.382 (0.34, 0.42)	0.315 (0.295, 0.33)	ASD: *F* = 4.8, *p* = 0.032; Age: *F* = 3.6, *p* = 0.062
17-Hydroxyprogesterone/17-hydroxypregnenolone sulfate		0.173 (0.14, 0.21)	0.102 (0.091, 0.12)	ASD: *F* = 11.4, *p* = 0.001; Age: *F* = 0, *p* = 0.93
*AKR1C1/HSD17B2 (20α-hydroxy-/20-oxo-steroid)*				
20α-Dihydropregnenolone/pregnenolone		0.673 (0.59, 0.76)	0.857 (0.8, 0.92)	ASD: *F* = 5.6, ***p*** = **0.021**; Age: *F* = 0.6, *p* = 0.427
20α-Dihydropregnenolone/pregnenolone, sulfates		3.55 (3.28, 3.84)	3.92 (3.72, 4.14)	ASD: *F* = 2.2, *p* = 0.141; Age: *F* = 2.6, *p* = 0.111
5α-Pregnane-3α,20α-diol/allopregnanolone		4.43 (3.46, 5.62)	6.39 (5.52, 7.38)	ASD: *F* = 3.4, *p* = 0.069; Age: *F* = 3.3, *p* = 0.074
5α-Pregnane-3α,20α-diol/allopregnanolone, conjugates		3.78 (3.59, 3.98)	5.35 (4.99, 5.74)	ASD: *F* = 31.6, ***p*** < **0.001**; Age: *F* = 0, *p* = 0.947
5α-Pregnane-3β,20α-diol/isopregnanolone		2.3 (1.98, 2.66)	1.72 (1.38, 2.15)	ASD: *F* = 2.4, *p* = 0.13; Age: *F* = 4.2, *p* = 0.044
5β-Pregnane-3α,20α-diol/pregnanolone, conjugates		0.635 (0.58, 0.69)	0.798 (0.7, 0.9)	ASD: *F* = 4.5, ***p*** = **0.039**; Age: *F* = 1.9, *p* = 0.179
5α-Pregnane-3α,17,20α-triol/17-hydroxyallopregnanolone		28.1 (22.7, 34.3)	40.6 (30.9, 52.3)	ASD: *F* = 2.5, *p* = 0.126; Age: *F* = 2.1, *p* = 0.16
5β-Pregnane-3α,17,20α-triol/17-hydroxypregnanolone		9.11 (7.88, 10.5)	12 (9.77, 14.7)	ASD: *F* = 2.4, *p* = 0.124; Age: *F* = 0.3, *p* = 0.568
5β-Pregnane-3α,17,20α-triol/17-hydroxypregnanolone, conjugates		5.97 (5.3, 6.7)	8.56 (7.27, 10)	ASD: *F* = 6.4, ***p*** = **0.014**; Age: *F* = 22.4, ***p*** < **0.001**

Ale data are presented as means with 95% lower, upper 95% confidence intervals), only the variables with statistical significance up to *p* = 0.2 are shown. Bold values denote significant differences between the analysed groups.

*ASD* autism spectrum disorder, *CI* confidence interval, *CTR*L control.

**Fig. 2:**
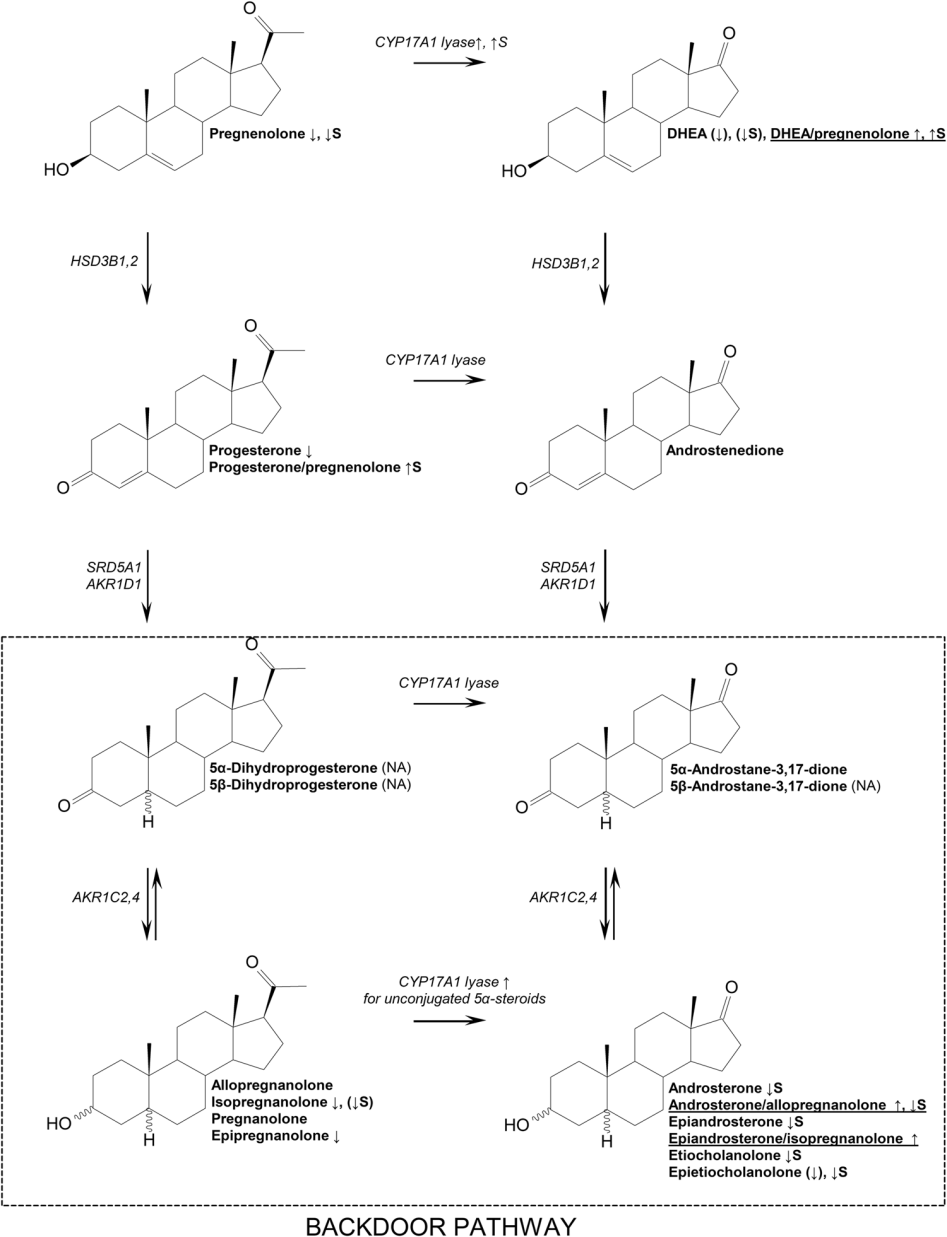
Simplified scheme illustrating differences between CTRL and ASD groups for CYP17A1 C17,20-lyase step. ↑/↓ denotes higher/lower steroid or steroid ratio in ASD group compare to control group; ASD, autism spectrum disorder; CTRL, control; S, sulfates; NA, hormone not assessed; (↑/↓), non-significant difference ↑/↓ significant difference between CTRL and ASD in favor to ASD with *p* value up to *p* = 0.2 according to the results presented in Tables 1 and  2.

The product to precursor ratios (PPRs) for steroids converted by CYP17A1 C17,20-lyase (Table 2 and Fig. 2) exhibited significantly higher ratios in ASD for DHEA/pregnenolone (*F* = 5.2, *p* = 0.026), their sulfates (*F* = 7.4, *p* = 0.009), androsterone/allopregnanolone (*F* = 10.9, *p* = 0.002) and epiandrosterone/isopregnanolone (*F* = 6.8, *p* = 0.12). On the other hand, ratio of androsterone/allopregnanolone sulfates was significantly lower in ASD compare to CTRL (*F* = 4.3, *p* = 0.041).

Significantly different ratios of steroid conjugates to the corresponding unconjugated steroids (C/U), which may reflect the balance between sulfotransferase SULT2A1 on one side and sulfatase STS on the other one are shown in Table 2. The C/U was significantly higher in ASD for pregnenolone (*F* = 12.3, *p* < 0.001), 17-hydroxypregnenolone (*F* = 18.3, *p* < 0.001), 17,20α-dihydroxy-4-pregnene-3-one (*F* = 22.9, *p* < 0.001), allopregnanolone (*F* = 6.7, *p* = 0.012), isopregnanolone (*F* = 8.6, *p* = 0.005), 5α-pregnane-3α,17,20α-triol (*F* = 8.9, *p* = 0.006), but significantly lower for 5α-pregnane-3β,17,20α-triol (*F* = 10.9, *p* = 0.002), 5α-pregnane-3α,20α-diol (*F* = 6.9, *p* = 0.011), 5β-pregnane-3β,20α-diol (*F* = 10, *p* = 0.002), androsterone (*F* = 22.1, *p* < 0.001), and 5β-androstane-3α,17β-diol (*F* = 10.8, *p* = 0.002).

The PPRs ratios reflecting HSD3B2 activity in *zona fasciculata* showed significantly lower 17-hydroxyprogesterone/17-hydroxypregnenolone (*F* = 4.8, *p* = 0.032) and 17-hydroxyprogesterone/17-hydroxypregnenolone sulfate ratio (*F* = 11.4, *p* = 0.001) in ASD compare to CTRL.

Supplementary Fig. 1 illustrates the changes between CTRL and ASD in non-corticoid immunoprotective substances, while the relevant PPRs are shown in Table 2. The ratio of 7α-hydroxy-DHEA/DHEA, which may reflect the activity of CYP7B1 enzyme was significantly lower in ASD compare to CTRL (*F* = 13.8, *p* < 0.001), same the 7β-hydroxy-DHEA/DHEA ratio (*F* = 6.8, *p* = 0.012), which may reflect the same metabolic step as well as the CYP3A4 activity. Alternatively, the ratio of sulfated forms 5-androstene-3β,16α,17β-triol/androstenediol, reflecting most probably the activities of CYP3A4 and CYP7B1, was higher in ASD compare to CTRL (*F* = 14.1, *p* < 0.001). The activity of HSD11B1 enzyme tended to lower values in ASD compare to CTRL as indicated by the following ratios: 7β-hydroxy-DHEA/7α-hydroxy-DHEA (*F* = 13.8, *p* < 0.001), 5-androstene-3β,7β,17β-triol/5-androstene-3β,7α,17β-triol (*F* = 4.1, *p* = 0.047), and cortisol/cortisone (*F* = 4.4, *p* = 0.045). From the ratios probably affected by AKR1C3 activity only the ratio androstenediol/DHEA (*F* = 5.4, *p* = 0.24) was significantly lower in ASD compare to CTRL (*F* = 5.4, *p* = 0.024).

Table 2 shows significant PPRs which may reflect the activity of AKR1C1 (Supplementary Fig. 2) such as 20α-dihydropregnenolone/pregnenolone (*F* = 5.6, *p* = 0.021), conjugated 5α-pregnane-3α,20α-diol/allopregnanolone sulfate (*F* = 31.6, *p* < 0.001), 5β-pregnane-3α,20α-diol/pregnanolone (*F* = 4.5, *p* = 0.039), and 5β-pregnane-3α,17,20α-triol/17-hydroxypregnanolone (*F* = 6.4, *p* = 0.014). These PPRs indicate higher AKR1C1 activity in ASD group.

The association between ASD as a predicted variable and relevant predictors represented by unconjugated and conjugated steroids was evaluated by OPLS and multiple regression with the explained 14,6% variability of ASD risk with the highest reached correlation 26.8% in social interaction (Table 3).

**Table 3:** Relationships between degree of autism and predictors for the 1st predictive component as evaluated by O2PLS model (for details see Statistical analysis).

		O2PLS (Predictive component)				Multiple regression (dependent variables)														
						Asum			Bsum			Csum			SAsum			ADOSRawscore		
	Variable	Component loading	*t*-statistics	*R*^*a*^		Regression coefficient		*t*-statistics	Regression coefficient		*t*-statistics	Regression coefficient		*t*-statistics	Regression coefficient		*t*-statistics	Regression coefficient		*t*-statistics
Relevant predictors (matrix **X**)	Preg	0.250	8.59	0.820	**	0.035	6.26	**	0.033	4.52	**	0.017	3.60	**	0.017	3.60	**	0.017	3.60	**
	PregC	0.269	13.96	0.882	**	0.031	4.66	**	0.028	3.99	**	0.015	3.26	**	0.015	3.26	**	0.015	3.26	**
	Preg17	0.195	6.92	0.640	**	0.024	1.99	*	0.022	2.13	*	0.012	1.63		0.012	1.63		0.012	1.63	
	Preg17C	0.197	4.92	0.647	**	0.027	2.27	*	0.025	2.51	*	0.013	1.91	*	0.013	1.91	*	0.013	1.91	*
	Preg20a	0.245	8.46	0.803	**	0.029	4.88	**	0.027	4.29	**	0.014	3.38	**	0.014	3.38	**	0.014	3.38	**
	Preg20aC	0.275	24.17	0.901	**	0.033	4.64	**	0.031	3.73	**	0.016	3.88	**	0.016	3.88	**	0.016	3.88	**
	DHEA	0.275	27.71	0.902	**	0.034	6.28	**	0.032	4.88	**	0.016	4.41	**	0.016	4.41	**	0.016	4.41	**
	DHEAC	0.260	13.59	0.852	**	0.028	5.24	**	0.026	3.94	**	0.014	3.20	**	0.014	3.20	**	0.014	3.20	**
	DHEA7a	0.245	11.65	0.804	**	0.036	8.30	**	0.034	4.14	**	0.018	3.40	**	0.018	3.40	**	0.018	3.40	**
	AdiolC	0.274	24.24	0.899	**	0.039	7.13	**	0.037	3.96	**	0.019	4.25	**	0.019	4.25	**	0.019	4.25	**
	AT16aC	0.257	15.17	0.843	**	0.037	6.63	**	0.034	6.09	**	0.018	3.34	**	0.018	3.34	**	0.018	3.34	**
	A2	0.233	7.42	0.765	**	0.029	3.30	**	0.027	2.62	*	0.014	2.73	*	0.014	2.73	*	0.014	2.73	*
	P3b5a	0.226	5.14	0.748	**	0.038	6.10	**	0.036	4.42	**	0.019	3.33	**	0.019	3.33	**	0.019	3.33	**
	P3b5bC	0.245	10.48	0.802	**	0.029	4.93	**	0.027	4.98	**	0.014	2.84	*	0.014	2.84	*	0.014	2.84	*
	P3b5a20aC	0.273	42.67	0.895	**	0.038	5.44	**	0.036	4.13	**	0.019	4.17	**	0.019	4.17	**	0.019	4.17	**
	A5a	0.201	10.61	0.665	**	0.029	3.37	**	0.027	2.48	*	0.014	6.56	**	0.014	6.56	**	0.014	6.56	**
	A3a5a11bC	0.211	10.29	0.693	**	0.035	4.08	**	0.033	3.86	**	0.017	3.07	**	0.017	3.07	**	0.017	3.07	**
(matrix **Y**)	Asum	0.510	4.49	0.543	**	**Asum**														
	Bsum	0.476	4.25	0.333	**				**Bsum**											
	Csum	0.248	3.07	0.247	**							**Csum**								
	SAsum	0.492	5.13	0.412	**										**SAsum**					
	ADOSRawscore	0.493	5.23	0.342	**													**ADOSRawscore**		
Explained variability		14.6% (12.7% after a cross-validation)				28.5% (26.8% after a cross-validation)			10.5% (8.2% after a cross-validation)			6.0% (5.1% after a cross-validation)			17% (15.1% after a cross-validation)			10.9% (8.5% after a cross-validation)		

*Preg* pregnenolone, *Preg17* 17-hydroxypregnenolone, *Preg20a* 20α-dihydropregnenolone, *DHEA* dehydroepiandrosterone, *DHEA7a* 7α-hydroxy-DHEA, *Adiol* androstenediol, *AT16a* 5-androstene-3β,16α,17β-triol, *A2* androstenedione, *P3b5a* isopregnanolone, *P3b5b* epipregnanolone, *P3b5a20*a 5α-pregnane-3β, 20α-diol, *A5a* 5α-androstane-3,17-dione, *A3a5a11b* 11β-hydroxyandrosterone, *C* conjugate/sulfate; ADI-R subdomains: *Asum* social interatction, *Bsum* communication and language, *Csum* repetitive, restricted and stereotyped interests.

^a^*R* component loadings expressed as a correlation coefficients with predictive component

*p < 0.05, **p < 0.01.

The predictors in the OPLS model explained 18.4% of the variability (14.5% after cross-validation) of the variability in the prediction of ASD. The relationship between specific behavioral subdomains of ADI-R and ADOS-2 representing predictive variables and predictors represented by unconjugated and conjugated steroids were also evaluated by OPLS model (Supplementary Table 1).

## Discussion

To our knowledge, this is the first study describing the markers of steroidogenesis across the whole pathway in plasma of boys diagnosed with ASD and neurotypical CTRL children. Only few studies dealing with the whole cascade of steroidogenesis have been published to this date, as majority of reports focus mostly on the narrow selection of the hormones and the bigger picture might be missing^12,22^. The human steroidogenesis is strongly age-dependent, particularly in children and pubescents. Even if the children at the age of 6 have still negligible activity of adrenal *zona reticularis*^23^ some data indicate that adrenarche is a gradual process^24^. Despite that exclusively pre-pubertal, pre-school children were recruited, a strong effect of age on the steroidogenic activity was observed. Therefore, firstly, our statistical analyses were age-adjusted and secondly, the comparisons of our data with other studies examining older children are limited.

Gasser et al. detected unconjugated steroids in urine of pubertal boys with ASD. They observed significantly higher level of most of the measured androstanes like androsterone, etiocholanolone, androstenediol, 11β-hydroxyandrosterone, 11β-hydroxyetiocholanolone, DHEA, 5-androstene-3β, 17β-diol in ASD compared to CTRL. This fact might be explained by precocious adrenarche leading to premature puberty described even in individuals having ASD^13,25^. Gasser et al. analyzed steroid pathway in pubertal individuals, thus, sexual maturity have to be critically taken into account^12^. On the other hand, our results showed that sulfated androstanes like androsterone, epiandrosterone, etiocholanolone were lower in ASD group. This might be explained by the fact, that individuals recruited into our study were pre-pubertal boys while participants recruited into the study published by Gasser et al. were pubertal boys. Thus, the relation to the onset of the puberty mentioned above might represent a key factor in the comparison of these two studies.

Majewska et al.^13^ performed analysis of steroid hormones in saliva of autistic and control male and female children from two age groups, 3–4 and 7–9 years. They observed non-significantly higher level of pregnenolone and its sulfate in ASD compare to CTRL in the group of 3–4 years old boys. Concerning these substances, we observed similar results. In addition, they found lower levels of allopregnanolone and allopregnanolone sulfate in ASD in comparison with CTRL. Our results showed no significant differences in these hormones. Oppositely to our study, they also observed higher level of androsterone but also higher level of androsterone sulfate, etiocholanolone sulfate in 3–4 years old boys and epiandrosterone sulphate pointing to the changes in the alternative backdoor pathway of androgens production as well as higher activity of SULT2A1 in androgens.

We observed changes across the pathway from pregnenolone sulfate (lower in ASD), via progesterone toward 17-hydroxyprogesterone up to inactive cortisol metabolite corticosterone. Pregnenolone and its sulfate as neurosteroids play role in neurodevelopment and neural plasticity^26^. It is currently well known, that pregnenolone has anxiolytic effects^27^ and plays role in improvement of depression symptoms^28^. Sripada et al. showed that oral administration of pregnenolone could be associated with increased activation of neuronal circuits controlling emotion regulation^29^. Even oral pregnenolone was used for adult individuals with ASD in the treatment of irritability and social withdrawal symptoms. It has been suggested the role of pregnenolone in improving social functioning, attenuating sensory abnormalities, and cognitive deficits^30,31^. Since pregnenolone was found as ASD predictor based on OPLS model, there is a suggestion for its role as a biomarker or a therapeutic target.

Our results showed further changes in the pathway headed from progesterone via 20α–dihydroprogesterone, 17α-hydroxyallopregnanolone sulfates toward androsterone sulfate. At the same time, the unconjugated androsterone positively modulate type A GABA receptors and is neuroprotective like androstanediol^32^, the levels of which were not significantly different between analyzed groups. The line of these changes is involved in the alternative backdoor pathway of androgens formation^33^. The starting point of this pathway represents 17-hydroxyprogesterone^34,35^. Although it did not significantly differ between observed groups CTRL and ASD, predictive component analysis assessed by OPLS model revealed it as one of the ASD predictors. We did not find any study investigating progesterone/17-hydroxyprogesterone and autism-like behavior.

Except the lower conjugated epipregnanolone in ASD, levels of 5α/β-reduced-20-oxo pregnanes were not changed. On the other hand, lower concentrations of 20α-dihydrometabolites (Table 1) were observed. Moreover, we detected lower PPRs of the 20α-hydroxy-pregnanes to their 20-oxo-counterparts (Table 2) indicate suppressed AKR1C1 activity in ASD group. Whereas the 20α-hydroxy-metabolites of GABAergic 20-oxo-pregnanes exhibit lower activity on type A GABA receptors than the parent steroids, this finding may be of importance.

Going ahead, the next metabolite in the alternative backdoor pathway is androsterone. However, its cognate metabolites like androsterone sulfate, epiandrosterone sulfate, etiocholanolone sulfate, epietiocholanolone sulfate, conjugated 5α-androstane-3β,17β-diol, 5β-androstane-3β,17β-diol, 11β-hydroxyetiocholanolone were lower in ASD proposing potential accumulation of androsterone in ASD. It should be pointed out that the adrenal possesses active adrenal *zona reticularis* only in children entering adrenarche but not in younger age. Therefore, the steroidogenesis in ASD subjects before and after adrenarche may substantially differ. The above 5α/β-reduced C19 steroids affect the functioning of a number of ionotropic and nuclear receptors. While androsterone (similar to etiocholanolone and androstanediol) is a positive type A GABA modulator and is, therefore, a neuroprotective substance, the sulphates of androsterone and epiandrosterone are their antagonists. In addition, these steroid sulphates work similarly on glycine receptors. Furthermore, unconjugated 5α/β-reduced steroids generally negatively modulate T-Type VGCCs that participate in the transmission of pain and unconjugated etiocholanolone similarly act on capsaicin receptors. Finally, a number of unconjugated 5α/β-reduced pregnanes and androstanes bind to nuclear pregnane X-type receptors (PXRs), which are involved in the elimination of xenobiotics as well as endogenous toxic substances, including some steroids^36^.

Our data indicate that ASD children before adrenarche demonstrate upregulated CYP17A1 activity (due to absence of functional *zona reticularis*) but this augmentation is mainly limited to the C17-hydroxylase step. The reduced activity of C17,20-lyase step at reduced levels of pregnenolone and its much more abundant sulfate might be associated with higher substrate consumption in the C17-hydroxylase step. However, the extra-adrenal activity of the C17,20-lyase step should be also considered. Same, the association between AQ and Asperger syndrome and CYP17A1 was found. Moreover, the relation between CYP17A1 single nucleotide polymorphism and ASD was described^37^. Our data also indicate elevated sulfotransferase activity in ASD group based on the PPRs except androgens. The SULT2A1 is active in both *zona fasciculata* and *zona reticularis* even if the former adrenal zone is less active^38^. It seems that androsterone might play an important strategic point in relation to ASD as well. In contrast to adrenal *zona reticularis*, which does not function in early childhood, the *zona fasciculata* (lacking the CYB5 enzyme, which blocks the HSD3B2, boosts the SULT2A1 activity and elicits the C17,20-lyase activity of the CYP17A1 enzyme) readily converts 17-hydroxypregnenolone to 17-hydroxyprogesterone. Our data demonstrate suppressed activity of the HSD3B2 in the ASD group. These results highlight the effect of these steroids in the pathogenesis of ASD. Recent studies analyzing steroidogenesis as a complex of hormones point to a crucial role of hormone-converting enzymes^12,13^. Hypothetically detection of a candidate enzyme instead of hormone itself or its appropriate gene in sense of expression or polymorphism could be helpful in diagnostics.

Although androsterone represents a weak androgen, it acts as a positive allosteric modulator of GABA receptors^39^. Concentration of androsterone showed to be sex-dependent^34^, thus hypothetically it might contribute to the fact that ASD is more diagnosed in males. Despite that androsterone has weaker androgen activity in comparison with TST its role is irreplaceable during the developmental stage. Alternative backdoor pathway is strongly involved in the masculinization of fetus^34,40^. Androsterone was found to be the most abundant androgen of the alternative backdoor pathway present in the placenta, fetal liver and adrenal glands^34^. Abnormalities and disorders of the placenta and their relation to ASD have already been described, thus, it might be potentially related to the disruption of alternative backdoor pathway^41^. Moreover, it has been hypothesized that backdoor pathway might be responsible for extensive masculinization in women having congenital adrenal hyperplasia^42^. Disruption in the synthesis of these hormones leading to their higher expression was observed also in ovaries of women with polycystic ovary syndrome^43^. This condition was previously already associated with ASD pattern and ASD development^44,45^. Unfortunately, considerations regarding the changes in this pathway have not yet been taken into account while the male bias was discussed^4,46,47^. Assessment of hormones involved in the alternative backdoor pathway could serve as hint in pre-diagnostic process of ASD. Unfortunately, our findings currently generalize only pre-pubertal male individuals. Although hormones of alternative backdoor pathway are presented in females as well, their association with ASD pathogenesis has to be clarified^34^.

In our study, surprisingly, only few differences were observed between ASD and CTRL in favored group of androgens. It has been described that 7α-hydroxy-DHEA act as an anti-glucocorticoid^48^. Our results showed a relationship between DHEA, DHEAS, and androstenedione and both social skills deficits and restricted interest in ASD.

Current studies, however, remain to be inconclusive regarding the role of androgens in ASD etiology. Ruta et al. did not find any relationship between DHEA sulfate, total and free TST, and estradiol and ASD. However, regression analysis in their study showed that diagnosis predicted androstenedione levels, which were elevated in the serum in ASD^49^. On the other hand, El-Baz et al. found that hyperandrogenemia with higher levels of free TST, DHEA, and androstenedione increased with the autism severity^50^. DHEAS possess neuroprotective effect e.g., by supporting neurogenesis and neural survival by protecting against apoptosis. Moreover, its positive impact on memory has been described^51^. However, memory impairment in a sense of working memory or episodic memory has been observed in ASD individuals^52,53^. Except adrenal glands, DHEA/S is produced in the brain as well. Nevertheless, the production and metabolism of DHEA might vary between individual brain regions^51^. In the hippocampus, DHEA is further metabolized to 7α-hydroxy-DHEA, which lower level was observed in this study. Interestingly, differences in the hippocampal asymmetry and mass have been already reported in ASD^54,55^. Moreover, reduced hippocampal connectivity during memory retrieval related to ASD was recently observed^56^. Since this study demonstrated significantly lower levels of these hormones, their either measurement or targeted therapy could be applied into the practice. The 7α/β-hydroxy-, 16α-hydroxy- and 7-oxo-derivatives of C19 Δ5 steroids are effective immunoprotective agents that stimulate immune response on the one hand and suppress autoimmune processes on the other. In addition, the 7-oxygenated steroids act as so-called ergosteroids, activating the enzymes glycerol-3-phosphate dehydrogenase and a malic enzyme. The final effect of ergosteroids may be comparable to that of thyroid hormones^36^.

Concerning the alterations of 7 and 16-oxygenated metabolites of DHEA and androstenediol, our data (Table 2) indicate the attenuated activity of the CYP7B1 enzyme but intensified 16α-hydroxylation of androstenediol was probably catalyzed by CYP3A4 enzyme. While the CYP7B1 and CYP3A4 may inactivate the substrates for the synthesis of active sex hormones this finding may be associated with altered androgen levels of ASD patients. Androstenediol represents a direct metabolite of DHEA having immunomodulating effect together with an ability to stimulate immune response of the organism to viral agents^57,58^. A lower concentration of endogenous steroids, including DHEA was found to be related to autoimmune diseases e.g., rheumatoid arthritis, multiple sclerosis or inflammatory bowel disease^59–61^. Higher prevalence and susceptibility to autoimmune diseases, inflammation, and abnormal immune reaction are commonly associated with ASD^62,63^. Our results completed with the results and observations from other studies show the complexity of ASD pathogenesis.

Distribution of ASD between males and females shows us that there must be a risk factor associated with being a male presented in the etiopathogenesis of ASD. However, looking only on TST might not fully explain these differences. This is the first evidence of changes in alternative backdoor pathway in synthesis of androgens in plasma of individuals with ASD. However, it seems that more than a hormonal change stands behind the pathology of ASD and altered hormonal profile might be a result of altered gene expression of hormones-associated or related genes^64^. For this reason, steroid hormonal profile as well as genetic background with focusing on the alternative backdoor pathway of pre-pubertal girls, pubertal girls and boys, individuals during early and late adulthood and adults of both genders with ASD having various ASD severities should be further investigated. Moreover, ASD children included into this study were pre-pubertal children with very low communication skills and no phrase speech. Whether verbal individuals diagnosed with less severe ASD symptoms would share the same trend within the hormonal profile is questionable. However, the increased level of androgens positively correlated with the severity of ASD^50^, thus altered alternative backdoor pathway of androgens synthesis might be expected too. Confirmation of the observed changes in other subgroups and modules of ASD individuals could lead to diagnostic profiling and formation of diagnostic panel composed of groups of steroids, converting enzymes and genes^65^. Standardized diagnostic approaches would emphasize diagnostic power of profiling by behavioral description and application of selected diagnostic scores with predictive variables.

The main limitation of this study is a missing evaluation of cholesterol as a precursor for steroid hormones synthesis. Unfortunately, some of the major hormonal steroids being the important intermediates and products like 5α-dihydrotestosterone, which would have emphasized our findings, were not detected. Unfortunately, we did not detected mineralocorticoids like aldosterone using the GC-MS/MS due to the chemical nature of these molecules^21^. Another limitation might be that control children in the control group did not undergo the diagnostic procedure ADOS-2 and ADI-R.

In conclusion, in spite of scarce differences in circulating steroids between ASD and CTRL group, our data obtained from boys in early childhood indicate augmented activities of CYP17A1 hydroxylase step and SULT2A1 at reduced HSD3B2 activity in ASD group. This finding is consistent with the results reported in older children, in whom the adrenal *zona reticularis* significantly influences the steroid levels. Furthermore, we detected the suppressed activity of CYP7B1 enzyme readily metabolizing the precursors of sex hormones on one hand but increased anti-glucocorticoid effect of 7α-hydroxy-DHEA via competition with cortisone for HSD11B1 on the other hand. Analyses of the steroidogenesis revealed changes in those hormones involved in the alternative backdoor pathway of androgens production. This finding might be considered as a crucial since only changes in dominant metabolites were believed to contribute to ASD development. Looking for the origin of these differences and changes might reveal the potential biomarkers and shed a light into the ethiopathogenesis of autism.

## Supplementary information

Supplementary Figure legends

Supplementary Table legends

Supplementary Table 1

Supplementary Figure 1

Supplementary Figure 2
