# Supplementary material for: Alteration of the steroidogenesis in boys with autism spectrum disorders
Source: Transl Psychiatry. 2020 Oct 6;10:340. doi: 10.1038/s41398-020-01017-8 (PMC7538887; doi:10.1038/s41398-020-01017-8)
Supplement: Supplementary file 1 — Supplementary Figure legends [file 41398_2020_1017_MOESM1_ESM.docx]

Manuscript Number: 2019TP000907RR

Title: Alteration of the steroidogenesis in boys with autism spectrum disorders

Authors: Katarina Jansakova, Martin Hill, Diana Celarova, Hana Celusakova, Gabriela Repiska, Marie Bicikova, Ludmila Macova, and Daniela Ostatnikova

Supplementary Figure 1: Simplified scheme illustrating differences between CTRL and ASD groups in formation of non-corticoid immunomodulatory and immunoprotective steroids; **↑/ ↓** denotes higher/lower steroid or steroid ratio in ASD group compare to control group; C – conjugate, S – sulfates, NA – hormone not assessed, (↑/↓) – non-significant difference ↑/↓ significant difference between CTRL and ASD in favor to ASD with p value up to p=0.2 according to the results presented in Table 1 and Table 2

Supplementary Figure 2: Simplified scheme illustrating differences between CTRL and ASD groups for AKR1C1; **↑/ ↓** denotes higher/lower steroid or steroid ratio in ASD group compare to control group; C – conjugate, S – sulfate, NA – hormone not assessed, (↑/↓) – non-significant difference ↑/↓ significant difference between CTRL and ASD in favor to ASD with p value up to p=0.2 according to the results presented in Table 1 and Table 2
