# Supplementary material for: Alteration of the steroidogenesis in boys with autism spectrum disorders
Source: Transl Psychiatry. 2020 Oct 6;10:340. doi: 10.1038/s41398-020-01017-8 (PMC7538887; doi:10.1038/s41398-020-01017-8)
Supplement: Supplementary file 2 — Supplementary Table legends [file 41398_2020_1017_MOESM2_ESM.docx]

Manuscript Number: 2019TP000907RR

Title: Alteration of the steroidogenesis in boys with autism spectrum disorders

Authors: Katarina Jansakova, Martin Hill, Diana Celarova, Hana Celusakova, Gabriela Repiska, Marie Bicikova, Ludmila Macova, and Daniela Ostatnikova

Supplementary Table 1. Relationships between ASD and individual steroids as predictors for the 1st predictive component evaluated by OPLS model; LLR – logarithm of likelihood ratio – logarithm of the ratio of the probability that the subject is ASD or CTRL; aR - component loadings expressed as a correlation coefficients with predictive component, *p<0.05, **p<0.01
