# Supplementary material for: Alteration of the steroidogenesis in boys with autism spectrum disorders
Source: Transl Psychiatry. 2020 Oct 6;10:340. doi: 10.1038/s41398-020-01017-8 (PMC7538887; doi:10.1038/s41398-020-01017-8)
Supplement: Supplementary file 3 — Supplementary Table 1 [file 41398_2020_1017_MOESM3_ESM.docx]

Supplementary Table 1.

|  |  | **OPLS** (Predictive component) | | | | **Multiple regression** | | |
| --- | --- | --- | --- | --- | --- | --- | --- | --- |
|  | **Variable** | Component loading | t-statistics | R*^a^* |  | Regression coefficient | t-statistics | |
| Relevant predictors  (matrix **X**) | Pregnenolone | -0.402 | -21.84 | -0.845 | ** | -0.069 | -3.23 | ** |
|  | 7α-Hydroxy-DHEA | -0.298 | -6.99 | -0.628 | ** | -0.062 | -5.08 | ** |
|  | Progesterone | -0.338 | -9.45 | -0.711 | ** | -0.071 | -3.37 | ** |
|  | 20α-Dihydroprogesterone | -0.323 | -17.04 | -0.679 | ** | -0.056 | -2.61 | * |
|  | 17-Hydroxyprogesterone | -0.270 | -4.10 | -0.568 | ** | -0.064 | -2.42 | * |
|  | 17,20α-Dihydroxy-4-pregnen-3-one | -0.381 | -8.31 | -0.803 | ** | -0.065 | -4.22 | ** |
|  | 16α-Hydroxyprogesterone | -0.190 | -3.14 | -0.399 | ** | -0.065 | -3.06 | ** |
|  | Isopregnanolone | -0.387 | -17.97 | -0.819 | ** | -0.064 | -2.72 | * |
|  | Conjugated 5α-pregnane-3β,17,20α-triol | -0.327 | -9.76 | -0.687 | ** | -0.061 | -2.26 | * |
|  | Conjugated 5β-androstane-3α,17β-diol | -0.248 | -5.67 | -0.452 | ** | -0.067 | -4.55 | ** |
| (matrix **Y**) | ASD_LLR | 1.000 | 2.94 | 0.429 | * |  | | |
| **Explained variability** | | 18.4% (14.5% after cross-validation) | | | | | | |
