# Supplementary figures and images for: Alteration of the steroidogenesis in boys with autism spectrum disorders
Source: Transl Psychiatry. 2020 Oct 6;10:340. doi: 10.1038/s41398-020-01017-8 (PMC7538887; doi:10.1038/s41398-020-01017-8)

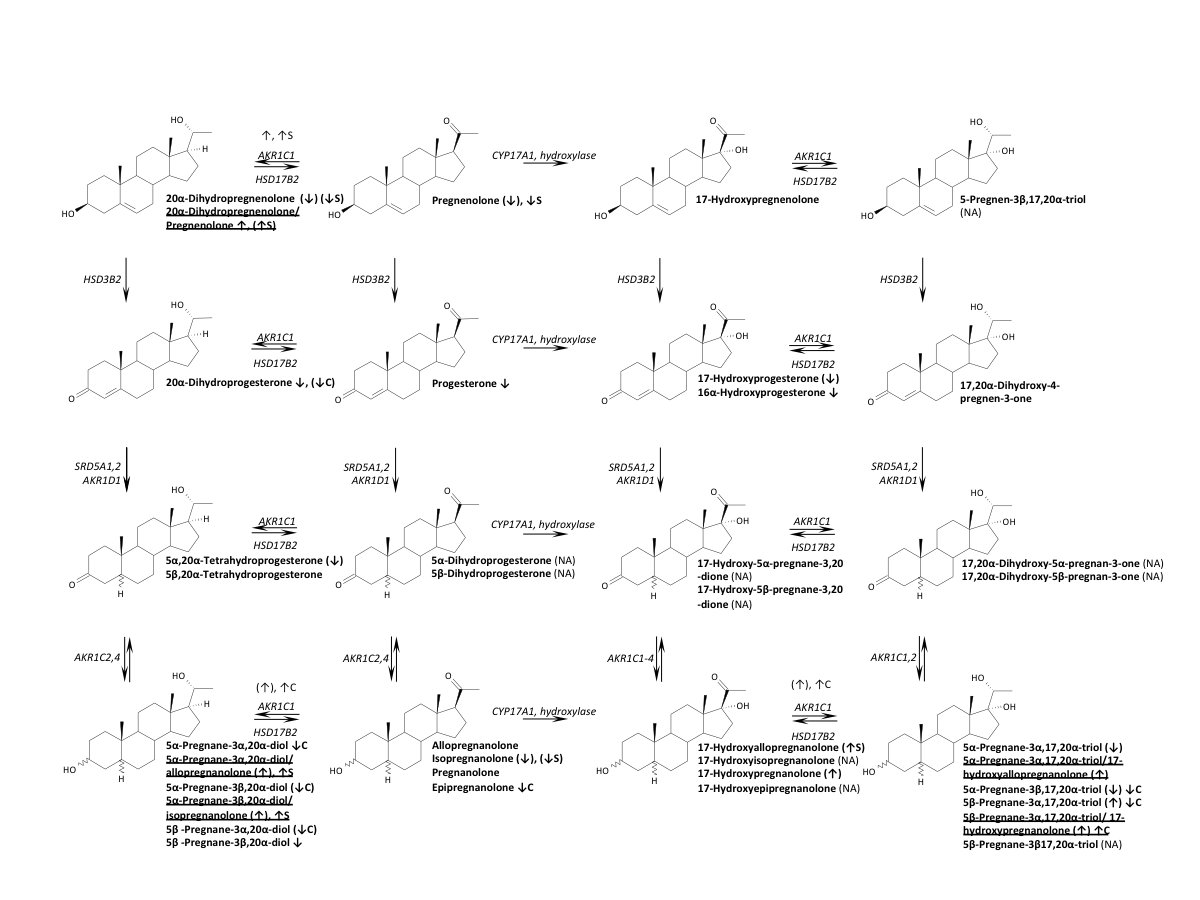

Supplement: Supplementary file 5 — Supplementary Figure 2 [file 41398_2020_1017_MOESM5_ESM.tif]
